# Comparative Analysis of MicroRNA Expression Profiles Between Skeletal Muscle- and Adipose-Derived Exosomes in Pig

**DOI:** 10.3389/fgene.2021.631230

**Published:** 2021-05-31

**Authors:** Weite Li, Shulei Wen, Jiahan Wu, Bin Zeng, Ting Chen, Junyi Luo, Gang Shu, Song-bo Wang, Yongliang Zhang, Qianyun Xi

**Affiliations:** ^1^Guangdong Province Key Laboratory of Animal Nutritional Regulation, College of Animal Science, South China Agricultural University, Guangzhou, China; ^2^Center for Proteomics and Metabolomics, State Key Laboratory of Biocontrol, School of Life Sciences, Sun Yat-sen University, Guangzhou, China; ^3^National Engineering Research Center for Breeding Swine Industry, College of Animal Science, South China Agricultural University, Guangzhou, China

**Keywords:** pig, muscle, adipose, exosome, microRNA

## Abstract

Skeletal muscle and adipose tissues are both involved in regulation of metabolism. In the skeletal muscle-adipose tissue crosstalk, exosomes may play an important role but the main components of exosomes are not clear. In this study, we found skeletal muscle-derived exosomes can inhibit adipogenesis of porcine preadipocytes. We identified microRNA expression profiles of muscle exosomes and adipose exosomes by high-throughput sequencing. There were 104 (both novel and known microRNAs) microRNAs differentially expressed (DE miRNAs) between M-EXO (muscle-derived exosomes) and A-EXO (adipose–derived exosomes) groups. A total of 2,137 target genes of DE miRNAs for M-EXO and 2,004 target genes of DE miRNAs for A-EXO were detected. Bioinformatic analyses revealed that some DE miRNAs of M-EXO (especially miR-221-5p) were mainly enriched in lipid-related metabolism processes. The findings may serve as a fundamental resource for understanding the detailed functions of exosomes between the skeletal muscle-adipose crosstalk and the potential relationship between skeletal muscle atrophy and obesity.

## Introduction

Muscle cell and adipose cell both belong to the mesodermal cell lineage. This same origin implies there may exist a special interaction between muscle and adipose. Fat ectopic accumulation can cause muscle atrophy (Teng and Huang, 2019). Chronic high fat feeding impairs the ability of murine skeletal muscle to cause hypertrophy, and then muscle hypertrophy accelerates white adipose tissue to brown and decreases adipose tissue deposition (Sitnick et al., 2009). Studies has shown that proteins of Wnt family may play an important role in muscle-to-adipose interaction, and Wnt/β-catenin signal transduction promotes growth of muscle cells and inhibition of intramuscular fat synthesis. This leads to muscle building and fat loss (Luo et al., 2008). Aydin et al. (2014) reports that irisin, a kind of myokines secreted by muscle tissue, could convert white adipose tissue into brown adipose tissue, enhancing energy expenditure. Leptin directly induces fatty acid oxidation (FAO) in skeletal muscle by AMPK pathway (Koo et al., 2019). A study has demonstrated adiponectin increases glucose uptake, enhances mitochondrial oxidation and modulates lipoclastic capacity via coupling fibroblast growth factor 21 (*FGF21*) actions from adipocytes to skeletal myocytes (Lin et al., 2013).

MicroRNAs (miRNAs) are also involved in the crosstalk between muscle and adipose tissues. MiRNAs are endogenous small non-coding RNAs (18–25 nucleotides) that post-transcriptionally regulate gene expression (Almeida et al., 2011). Some miRNAs, such as miR-222, miR-195, miR-103, and miR-10b are found to regulate glucose metabolism in muscle cells (He et al., 2007; Guay and Regazzi, 2017). Microvesicle-shuttled *miRNA-130b* is found to suppress adipogenesis and fat deposition in recipient adipocytes by targeting peroxisome proliferator-activated receptor-gamma (PPAR-γ) (Indrakusuma et al., 2015). More and more studies have shown that miRNAs are very important in the interaction between muscle cells and adipose cells.

In the last decade, exosomes are found to transport proteins, mRNAs, miRNAs to recipient cells (Kosaka et al., 2010). They are firstly identified in reticulocytes and were originally thought to be involved in the selective excretion of cellular waste (Colombo et al., 2014; Sagini et al., 2018). These small vesicles (50 to 150 nm) are related to endosomal pathway and are released in the extracellular space via merging multivesicular bodies (MVBs) from the cell membrane. Environmental stressors, disease and cell type can impact the sort of exosomal miRNA cargo, which suggest an active sorting and metabolic mechanism (Wahlgren et al., 2016).

Until now, there were few reports about exosomes from muscle (M-EXO) and adipose (A-EXO). Our study is aimed to identify miRNA profiles in M-EXO and A-EXO, and explore which miRNAs in M-EXO and A-EXO may be involved in communication of muscle and adipose tissues.

## Manuscript Formatting

### Materials and Methods

#### Ethical Approval

All the animal experiments contained in the article were conducted by Institutional Animal Care and Use Committee (IACUC) of South China Agricultural University.

#### Animals and Sample Collection

Four healthy 5-day-old piglets were selected from Guangzhou thoroughbred farm (Guangzhou, Guangdong, China) and exsanguinated by electric stunning. Longissimus dorsi muscle tissues and subcutaneous adipose tissues were dissected and transported to the laboratory, and transferred to DMEM-F12 medium (Gibco, New York, NY, United States).

#### Culture and Induction of Primary Porcine Preadipocytes

The preadipocytes were acquired according to our previous study (Wu et al., 2016). First of all, four samples of subcutaneous adipose tissue were cut into sections of 1 mm^3^ and transferred to DMEM-F12 medium. Minced tissues were digested with 0.2% type-II collagenase (Gibco, New York, NY, United States) for 2 h at 37°C with shaking. Then the digested tissues were filtered through a 150 μm mesh, and the filtrates were centrifuged at 600 *g*, 10 min. The pellets were resuspended by erythrocyte lysis buffer (Sangon Biotech, Shanghai, China) and stood for 10 min to lyse erythrocytes. Then the mixture was centrifuged at 800 *g*, 10 min. Subsequently, the pellets were resuspended with DMEM-F12. The resuspended liquids were filtered through a 40 μm mesh and then centrifuged at 800 *g*, 5 min. The pellets containing preadipocytes were resuspended and cultured in DMEM-F12 medium with 10% fetal bovine serum (FBS, Gibco, New York, NY, United States) at 37°C, 5% CO_2_. The preadipocytes were induced to mature adipocytes with an induction medium (10% FBS, DMEM-F12, 50 μM oleic acid, 0.5 M Octoic acid, 50 nM insulin, 50 nM dexamethasone). The first day of induction was designated as Day 0. In the induction, 10 μg exosomes were added to per well at Day 0 and treated for 24 h.

#### Culture of Skeletal Muscle Satellite Cells

The skeletal muscle satellite cells were obtained as described in our previous reports (Wang et al., 2012). Four muscle samples were cut into small pieces and transferred to DMEM-F12. The minced tissues were digested for 1 h with 0.2% type-II collagenase (Sangon Biotech, Shanghai, China). Then the digested tissues were centrifuged at 1,500 *g*, 4°C, 10 min. The pellets were resuspended in DMEM-F12 and centrifuged at 800 *g*, 4°C, 10 min for 3 times. Then cell resuspension solutions were filtered through a 200 mm cell strainer. The filtrated supernatants were centrifuged at 800 *g*, 4°C, 5 min. The underlying pellets were resuspended in DMEM-F12 medium and incubated in a cell culture flask at 37°C, 5% CO_2_ for 1 h. The fibroblasts were quickly adhered to the bottom of cell culture flask, whereas the skeletal muscle satellite cells remained in the supernatant. Finally, the skeletal muscle satellite cells were cultured in DMEM-F12 (10%FBS) at 37°C, 5% CO_2_.

#### Isolation of Exosomes

After reaching 80% confluency (about 6 × 10^6^ cells), cells were washed with phosphate buffer saline (PBS, Sangon Biotech, Shanghai, China) three times and incubated with fresh DMEM-F12 medium for 48 h. The supernatant was collected and centrifuged at 1,500 *g* for 15 min to remove dead cells and cell debris and mixed with ExoQuick precipitation solution (System Biosciences, Palo Alto, CA, United States) at an 1:1 ratio and incubated overnight at 4°C, and then the mixture was centrifuged at 1,500 *g*, 4°C, 30 min to precipitate exosomes. The supernatant was removed carefully, and then the pellet containing exosomes was resuspended in PBS and stored at −80°C. BCA Protein assay kit (Bioteke, Beijing, China) was used to determine protein concentration of exosome.

#### Western Blot Assay

Western blot was performed to identify exosome special marker. Cell and exosome sample were lysed by RIPA (Solarbio, Beijing, China). Equivalent amounts of protein were separated by 10% SDS-PAGE and the samples were transferred onto PVDF membranes (Bio-Rad, CA, United States). The proteins were reacted with follow primary antibodies: rabbit anti-Alix (Sangon Biotech, Shanghai, China, D262028) and rabbit anti-TSG101 (ZenBio, Chengdu, China, 381538). Blocked with Blocking Buffer (NCM biotech, Suzhou, China, P30500) and incubated with the primary antibody overnight at 4°C. Then incubated with the secondary antibody for 1 h at room temperature.

#### Oil Red O Staining

On induction day 8, adipose cells were harvested and rinsed with PBS twice. Then they were fixed in 4% polyoxymethylene for 30 min at room temperature. Afterward the cells were stained with oil red O solution (Sangon Biotech, China) for 1 h at room temperature. The stained samples were washed by PBS and photographed with microscope (Nikon, Tokyo, Japan).

#### TG Assay

Cells were washed with PBS twice and then 1 ml PBS was added to each well. Ultrasonication of cells was performed by ultrasonic processor (Scientz, Ningbo, China). The products were centrifuged at 7,000 *g*, 4°C for 1 min. The supernatants were analyzed by TG assay by Triglyceride Assay Kit (Abcam, United Kingdom). TG level was normalized by total protein level, which was measured by BCA assay (Bioteke, Beijing, China).

#### Small RNA Library Construction and RNA Sequencing

Trizol Reagent (Invitrogen, Carlsbad, CA, United States) was used to extract total RNAs according to previous protocol (Rio et al., 2010). A total of 2 μg RNAs of each sample was collected to prepare the miRNA sequencing library using NEBNext^®^ Multiplex Small RNA Library Prep Set for Illumina^®^ (NEB, Ipswich, MA, United States). Briefly, T4 RNA ligase 1 and T4 RNA ligase 2 (truncated) were used to ligate adapters to the 3′ and 5′ ends of RNAs. Then RNAs were reverse transcribed to cDNA and amplified by PCR. Subsequently, the amplification products were purified on polyacrylamide gel electrophoresis. The library was denatured as single-stranded DNA molecules, captured on Illumina flow cells, amplified *in situ* as clusters and finally sequenced applied 50 cycles on Illumina HiSeq sequencer at Cloud-Seq Biotech (Shanghai, China).

#### Bioinformatics Analysis

Raw data were generated after sequencing, image analysis, base calling and quality filtering on Illumina sequencer. Firstly, Q30 (Q30 content represents the percentage of bases with Phred value greater than 30 in the total base) was used to perform quality control. The adaptor sequences were trimmed by cut adapt software (v1.9.3) (Martin, 2011). Then, trimmed reads from all samples were collected, and miRDeep2 software (v2.0.0.5) (Friedlander et al., 2012)was used to predict novel miRNAs. The trimmed reads were aligned to the merged pig pre-miRNA databases (known pre-miRNA from miRbase (v21) (Kozomara et al., 2018) plus the newly predicted pre-miRNAs) using Novoalign software (v3.02.12) with at most one mismatch. The numbers of mature miRNA mapped tags were defined as the raw expression levels of that miRNA. The read counts were normalized by TPM (tag counts per million aligned miRNAs) approach. Differentially expressed (DE) miRNAs between M-EXO and A-EXO groups were filtered through Fold change and false discovery rate (FDR) in OmicShare Tools^1^. Then, top DE miRNAs were chosen to predict their target genes using popular miRNA target prediction software (TargetScan v6, miRanda) (Enright et al., 2003; Grimson et al., 2007). Among all target genes, we chose the top 100 targets (Ranked from high miRanda structure score to low structure score) to map the miRNA-gene network. All targets were chosen if less than 100. MiRNA-target networks were plotted by cytoscape software (v2.8.0) (Betel et al., 2010; Smoot et al., 2010), while the Gene Ontology (GO) (Huang et al., 2008) and Kyoto Encyclopedia of Genes and Genomes (KEGG) (Minoru and Susumu, 2000) pathway analysis were performed based on the differentially expressed miRNAs.

#### Real-Time RT-PCR Analysis

Total RNA was reverse-transcribed into cDNA by MLV reverse transcriptase (Promega, M1701-10000U). RT-PCR analysis was performed on Agilent stratagene Mx3005P (Agilent, United States) with the Go Taq qPCR Master Mix (Promega, Madison, WI, United States). All reactions were run in triplicate. The cycle threshold (Ct) method was used to calculate expression values. Ct values were normalized to the reference gene (*U6* for miRNA, GAPDH for mRNA) as an endogenous control. The information of primers was listed in Supplementary Table 1.

#### Statistical Analysis

Statistical analyses were achieved by the SPSS software (v20). Differences between groups were analyzed by independent two-sample *t*-test. *P* < 0.05 indicates the difference is statistically significant.

### Results

#### M-EXO Inhibit Proliferation and Adipogenesis in Porcine Preadipocytes

**FIGURE 1:**
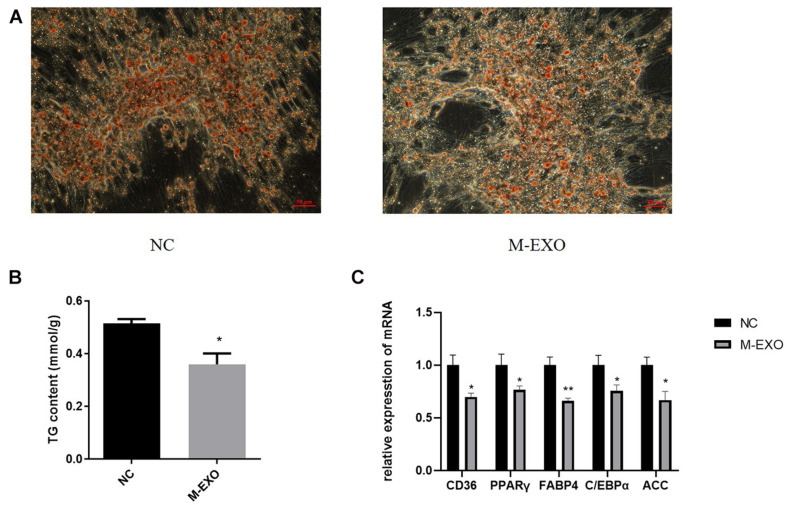
Muscle derived exosomes inhibited adipogenesis in porcine preadipocytes. **(A)** Oil Red O staining at induction day 8. NC: Treated with PBS Exosome: Treated with muscle exosome. Scale bars, 50 μm. **(B)** Triglyceride Assay was performed at induction day 8. TG level was adjusted by protein content. Each sample was assayed in duplicate (*n* = 6), *P < 0.05; **P < 0.01. **(C)** mRNA expression levels of CD36, PPARγ, FABP4, C/EBPα, and ACC. NC: Treated with PBS Exosome: Treated with muscle exosome (*n* = 6), *P < 0.05; **P < 0.01.

First at all, Exosome special protein markers Alix and TSG101 were identified by Western blotting to prove the accuracy of exosome (Supplementary Figure 1). Then we treated adipose cells with M-EXO. At Day 8 of induction, M-EXO significantly suppressed adipogenesis of adipocytes (Figure 1A). Furthermore, TG assay demonstrated that adipogenesis was significantly suppressed by M-EXO treatment (Figure 1B). The expression levels of various adipocyte markers were decreased when treated with M-EXO (Figure 1C). Together, these findings indicate that muscle exosomes inhibited adipogenesis of porcine preadipocytes.

#### Differentially Expressed miRNAs Profiles

A total of 5,634,600 and 4,938,001 raw reads (≥15 nt) were obtained from the A-EXO and M-EXO libraries, respectively. After removing contaminant reads (adaptor sequences, rRNA, virus etc.), we obtained 3,553,685 (A-EXO) and 3,078,361 (M-EXO) reads for subsequent analyses. The raw data was uploaded to SRA database (PRJNA665545). The length of all reads (both M-EXO and A-EXO) were distributed in a range of 16∼30 nt.

**FIGURE 2:**
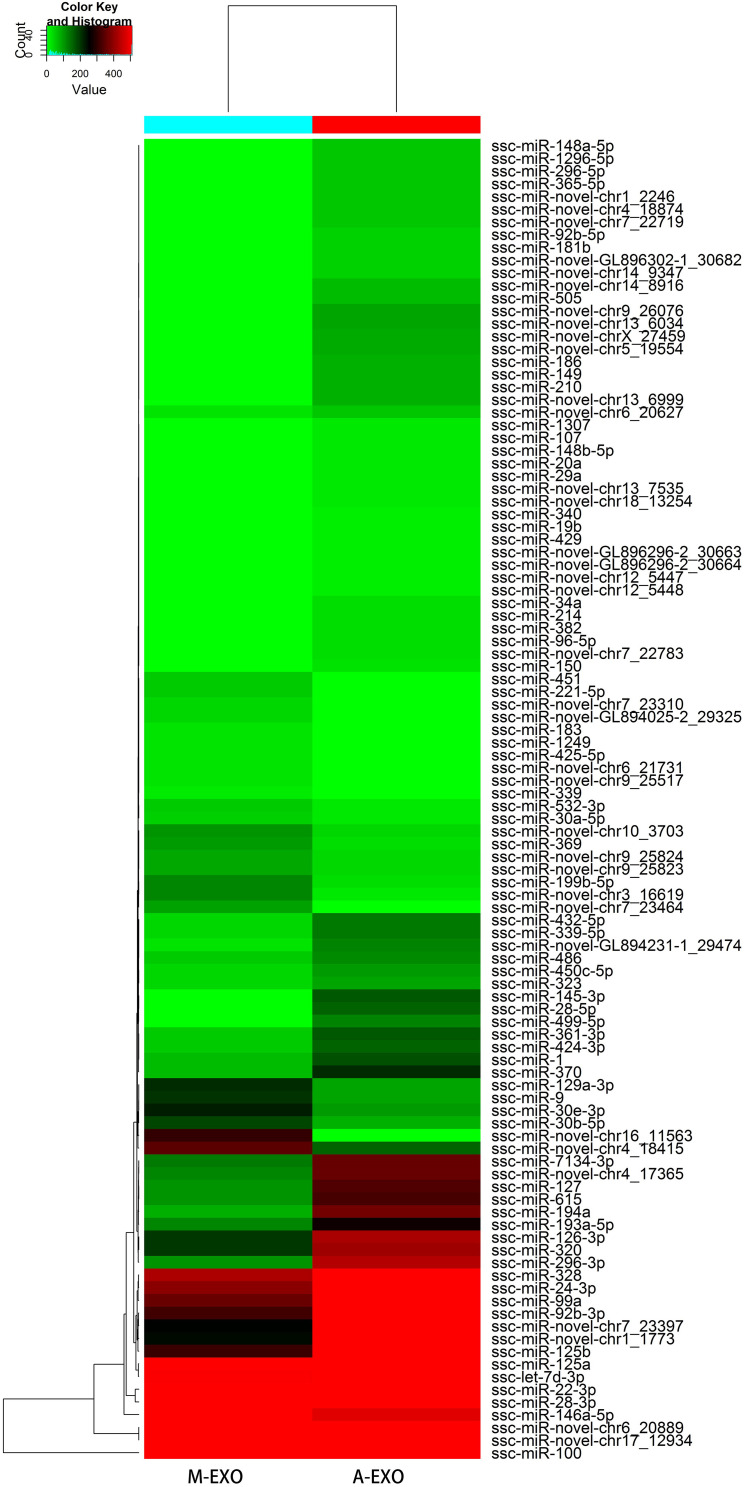
Heatmap of miRNAs in M-EXO and A-EXO group.

Consequently, a total of 191 miRNAs (52 novel miRNAs and 139 known miRNAs) were identified in A-EXO and M-EXO. There were 30 miRNAs counting more than 1000TPM (Transcripts Per kilobase Million) in M-EXO, and 40 miRNAs counting more than 1000TPM in A-EXO. Comparing all those identified miRNAs (both novel and known miRNAs) in A-EXO, 78 were downregulated and 26 were upregulated in M-EXO group, (Fold Change > 2) (Figure 2).

#### RT-PCR Validation of the Sequencing Data

**FIGURE 3:**
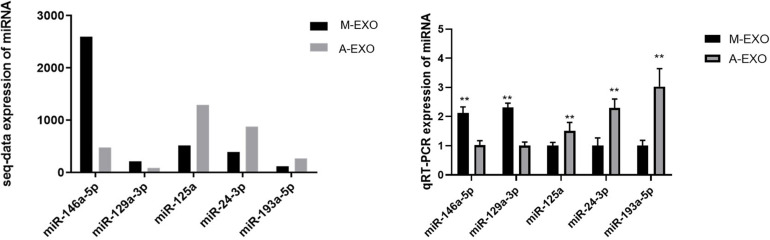
Validation of DE miRNAs by qPCR. Muscle-derived exosome: M-EXO; adipose-derived exosome: A-EXO. **P < 0.01. Left: Expression of miRNA of sequence data; Right: Expression of miRNA of qRT-PCR.

To validate the reliability of high-throughput sequencing data, RT-PCR were performed. Five miRNAs with different expression levels were selected randomly, of which *miR-146a-5p* and *miR-129a-3p* were upregulated in M-EXO, and *miR-125a*, *miR-24-3p, miR-193a-5p* were upregulated in A-EXO. The results showed that the regulation of those miRNAs expression was basically consistent with the miRNA-sequencing results (Figure 3). Next, we determine the 4 miRNAs expression in adipocytes after the treatment with M-EXO, miR-146a-5p upregulated significantly (Supplementary Figure 2).

#### Integrated Analysis and Functional Annotation

Target genes of the DE miRNAs were predicted to identify candidate biological processes in which the DE miRNAs may be involved in. In M-EXO group, a total of 2,137 genes were found to be potentially targeted by the most significantly DE miRNAs (*miR-183, miR-425-3p, miR-1249, miR-451, miR-146a-5p, miR-221-5p*), including *AKT2*, *IPPK*, *IRAK2*, which are associated with adipogenesis and lipogenesis. As for A-EXO group (*miR-28-5p, miR-145-5p, miR-149, miR-186, miR-499-5p*), 2,004 targets were detected, including *CRTC2, FOXO1, SLC2A4*, which are the key regulatory factors in glucose and lipid metabolism (Supplementary Figure 3).

**FIGURE 4:**
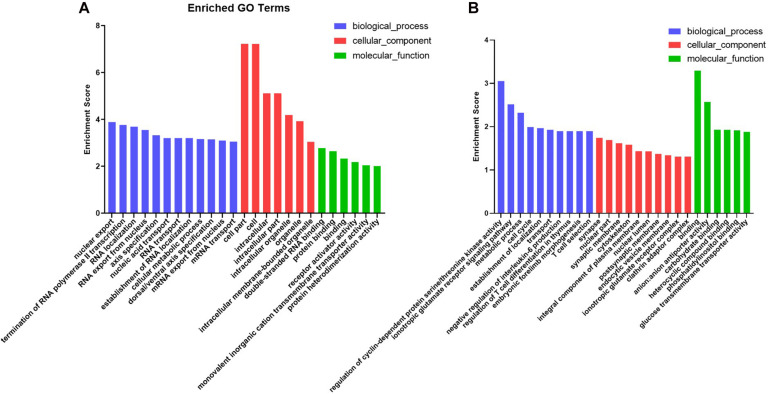
Enriched GO terms of genes targeted by the significant differentially expressed miRNAs. **(A)** Target genes of M-EXO (miR-183, miR-425-3p, miR-1249, miR-451, miR-146a-5p, miR-221-5p); **(B)** Target genes of A-EXO (miR-28-5p, miR-145-5p, miR-149, miR-186, miR-499-5p).

**FIGURE 5:**
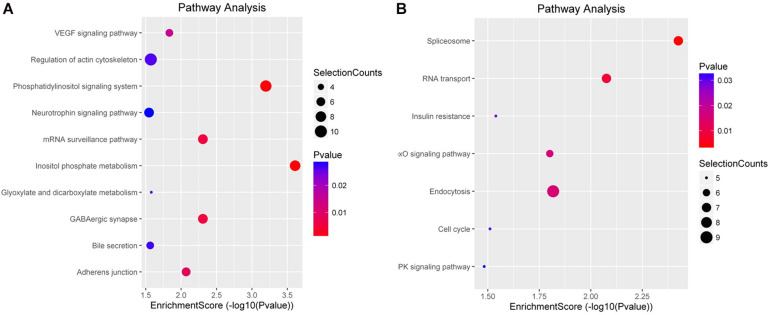
KEGG enrichment analysis of differentially expressed miRNAs. **(A)** Target genes of M-EXO (miR-183, miR-425-3p, miR-1249, miR-451, miR-146a-5p, miR-221-5p); **(B)** Target genes of A-EXO (miR-28-5p, miR-145-5p, miR-149, miR-186, miR-499-5p).

To characterize the regulation of those DE miRNAs, GO enrichment and KEGG pathway analyses were performed in this study. GO analysis showed that all those target genes in M-EXO group were enriched in many processes, such as RNA transport, nucleus mRNA export, protein binding, cellular metabolic process, receptor activator activity, and protein heterodimerization activity. In A-EXO group, they were enriched in regulation of cyclin-dependent protein serine/threonine kinase activity, glucose transmembrane transporter activity, phosphatidylinositol binding, and endocytic vesicle membrane (Figure 4). KEGG pathway analysis showed M-EXO group was enriched in inositol phosphate metabolism, phosphatidylinositol signaling system, mRNA surveillance pathway, GABAergic synapse, adherence junction, VEGF signaling pathway, glyoxylate and dicarboxylate metabolism, and actin cytoskeleton regulation (Figure 5). As for A-EXO group, target genes were enriched in spliceosome, FoxO signaling pathway, insulin resistance, and AMPK signaling pathway (Figure 5). Those data showed those miRNAs and their targets may associated with cell proliferation, carbohydrate metabolism, fat deposition, which all correlated with the crosstalk of muscle and adipose.

#### Differential Expression Between Tissues and Exosomes

**FIGURE 6:**
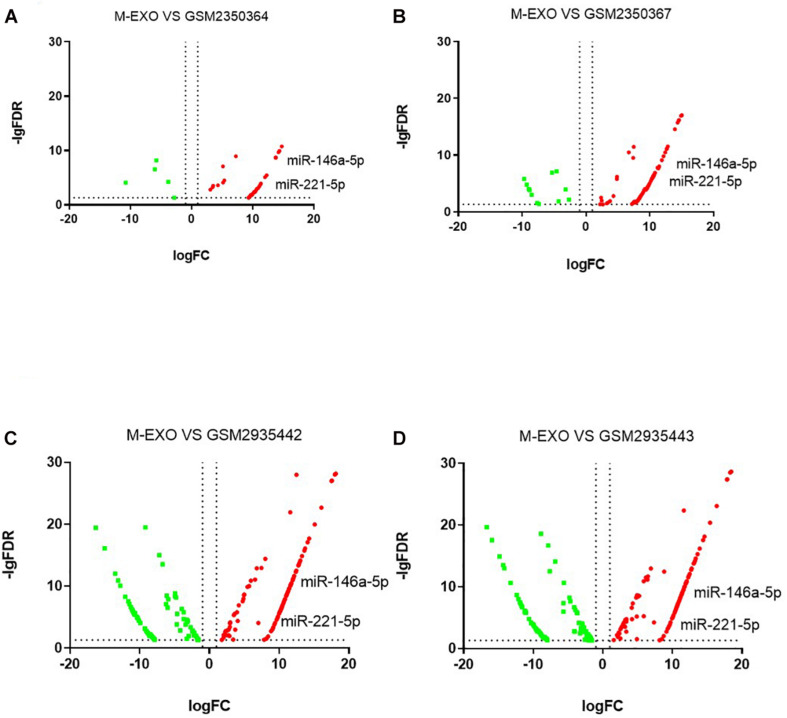
Comparison between M-EXO and MUS groups. **(A)** DE miRNAs in M-EXO vs. MUS (GSM2350364); **(B)** DE miRNAs in M-EXO vs. MUS (GSM2350367); **(C)** DE miRNAs in M-EXO vs. MUS (GSM2935442); **(D)** DE miRNAs in M-EXO vs. MUS (GSM2935443).

We downloaded four raw high-sequence data of pig muscle tissue (MUS) from NCBI GEO database). Then we examined miRNAs profiles in each MUS groups (GSM2350364, GSM2350367, GSM2935442, GSM2935443). Volcano plot showed DE miRNAs profiles of M-EXO as compared to MUS (Figures 6A–D). Compared with all four MUS groups, 31 miRNAs were upregulated in M-EXO, of which *miR-146a-5p* and *miR-221-5p* were upregulated in every M-EXO vs. MUS group (Figures 6A–D). Then, we filtered KEGG pathway of *miR-146a-5p* (Table 1) and *miR-221-5p* (Table 2), and data showed targets of *miR-221-5p* were enriched in adipocytokine signaling pathway, PI3K-Akt signaling pathway, FoxO signaling pathway, and insulin signaling pathway.

**TABLE 1:** Summary of KEGG pathways associated with miR-146a-5p.

**ID**	**Term**	***P*-value**	**Genes**
ssc04620	Toll-like receptor signaling pathway	0.000873	MAP3K8|IRAK1|TRAF6
ssc04010	MAPK signaling pathway	0.002261	MAP3K8|IRAK1|ERBB4|TRAF6
ssc03440	Homologous recombination	0.00292	RAD51B|RAD50
ssc05168	Herpes simplex virus 1 infection	0.004082	ZNF471|ZNF169|IRAK1|TRAF6

**TABLE 2:** Summary of KEGG pathways associated with miR-221-5p.

**ID**	**Term**	***P*-value**	**Genes**
ssc04920	Adipocytokine signaling pathway	0.001985	CPT1A|IKBKB|PRKAB1|G6PC3|TNFRSF1A
ssc04151	PI3K-Akt signaling pathway	0.002692	ITGA8|BRCA1|CCND1|CSF1|G6PC3|IKBKB|LAMC2|MCL1|COL4A1|LPAR2|FLT4
ssc04068	FoxO signaling pathway	0.005385	TGFB2|PRKAB1|CCND1|S1PR1|G6PC3|IKBKB
ssc04910	Insulin signaling pathway	0.024613	IKBKB|HKDC1|CRKL|G6PC3|PRKAB1

### Discussion

Both adipose tissue and skeletal muscle are recognized as endocrine organs secreting many bioactive factors, such as myokines and adipokines being involved in intercellular communication. Amongst these, myostatin (a myokine) is a negative regulator of muscle growth. Previous studies indicated depleting myostatin induces skeletal muscle hypertrophy and inhibits body fat accumulation (Argilés et al., 2005). Some previous articles also suggested erythropoietin as a myokine, Hojman et al. (2009) found overexpression of erythropoietin in obese mice resulted in a weight reduction. Adipokines, such as leptin and adiponectin are involved in the regulation of muscle (Carvalho et al., 2018). Recent findings showed exosome is an additional vehicle in intercellular communication (Guay and Regazzi, 2017). Exosome and exosomal miRNAs are new and more selective and specific approaches for the crosstalk in adipose tissue and muscle tissue. Exosomal *miR-130b* inhibits expression of *PGC-1*α in C2C12 myotubes (Wang et al., 2013). *MiR-200a* can block TCS1 expression and promote muscle hypertrophy (Fang et al., 2016).

We identified the DE miRNAs between M-EXO and A-EXO, of which 6 miRNAs were upregulated in M-EXO. Moreover, we compared the miRNAs profiles of M-EXO with 4 miRNA profiles of porcine muscle tissues (Downloaded from NCBI GEO). We found *miR-146a-5p* and m*iR-221-5p* were upregulated in M-EXO as compared to MUS groups. KEGG analysis showed *miR-146a-5p* and *miR-221-5p* were associated with MAPK signaling pathway, adipocytokine signaling pathway, PI3K-Akt signaling pathway, FoxO signaling pathway, and insulin signaling pathway. Studies have shown that activation of FoxO pathway inhibits lipogenesis through suppression expression of sterol regulatory element binding protein 1c (SREBP-1c) and glucokinase (Xiong et al., 2013), and activation of PI3K-Akt pathway promotes brown adipogenesis mediated by *GDF5* (Hinoi et al., 2014). Meerson et al. (2013) have also indicated that expression of adipose miR-221 is positively correlated with increasing BMI in the Pima Indian population. In human preadipocytes, proteomic analysis showed miR-221 overexpression upregulates several proteins (AKR1C1, FASN, and HADHB) involved in fat metabolism, mimicking activation of *PPAR* (Meerson et al., 2013). Associating those results with our data, we suggest that muscle derived exosomal *miR-221-5p* is probably associated with adipose tissue metabolism.

MiR-146a are potentially involved in adipocyte differentiation by targeting *C/EBP beta* and *Apo E* (Chartoumpekis et al., 2012). A study showed that *miR-146a-5p* inhibits TNF-α induced adipogenesis via targeting insulin receptor in primary porcine adipocytes (Wu et al., 2016). Wang et al. examined miRNA profiles of porcine muscle and adipose tissues in different developmental stage. Interestingly, *miR-146a-5p* is upregulated in adipose tissue on30d, 90d, 240d (Wang et al., 2017). This implies that *miR-146a-5p* may play an important role in development of adipose tissue. MiRNAs and exosomes can play important role in muscle tissue to other tissue communication (Guay and Regazzi, 2017; Lam et al., 2019). Thus we speculated part of *miR-146a* in adipose was derived from muscle cells via transportation of M-EXO. Exosomes from human skeletal myoblasts are shown to promote myogenesis of human adipose stem cells *in vitro* (Lam et al., 2019). Kuang et al. (2009) showed that *miR-146a* inhibits satellite cell differentiation via targeting Numb. The differentiation of C2C12 cells was rescued after inhibition of miR-146a (Kuang et al., 2009). Exosomal *miR-27a* can induce insulin resistance in skeletal muscle via repressing PPARγ (Yu et al., 2018). Zhou et al. (2010) found *miR-122, miR-323* and *miR-130a* were upregulated in longissimus muscle tissue of pigs at embryonic day 90. Our data showed that *miR-27a* (1.8 foldchange) and *miR-323* (2.2 foldchange) were upregulated in A-EXO. These results support previous findings and indicate that exosomes may serve as messenger between adipose tissue and muscle tissue.

Ropka-Molik et al. obtained miRNAs profiles between Pietrain and Hampshire breeds, muscle-specific *miR-206* was identified as DE miRNAs in Pietrain and Hampshire pigs differing in muscle weight (Katarzyna et al., 2018). They suggested that *miR-206* may play an important role in muscle growth and development. Inhibition of *miR-206* leads to skeletal muscle hypoplasia (O’Rourke et al., 2007). Deletion of *miR-206* in mice delays muscle regeneration induced by cardiotoxin injury (Liu et al., 2012). Muscle-specific *miR-206* is described to be involved in proliferation and differentiation (Lam et al., 2019). Chen et al. (2019) reported miR-1, miR-206 and miR133 family are DE miRNAs in skeletal muscle tissue of Guizhou miniature pig. But in our study, *miR-206* was not detected as a DE miRNA in M-EXO. The difference may be due to the mechanisms responsible for the upload of the exosomal miRNAs and difference of breed. The mechanisms remain largely unknown. Previous studies showed some miRNAs seemed to be sorted into exosomes preferentially, while others preferred to be retained in parental cells. Moreover, environmental stressors, cell type, and disease also contributed to sorting mechanism (Guay and Regazzi, 2017).

To the best of our knowledge, this is the first study that showed muscle derived exosome can attenuate proliferation and adipogenesis of preadipocytes as well as identified miRNAs profiles of porcine exosomes from muscle and adipose tissues. According to bioinformatic analyses, we suggested *miR-221-5p* and *miR-146a-5p* may serve as regulator in the muscle-adipose communication. However, the exact mechanism of how muscle-derived exosomal miRNAs affects adipose tissue remained to be determined in future study. This study may serve as a foundation for further studies on the detailed functions of exosomes between the skeletal muscle-adipose crosstalk and the potential relationship between skeletal muscle atrophy and obesity.

## Data Availability Statement

Publicly available datasets were analyzed in this study. This data can be found here: SRA database (PRJNA665545).

## Ethics Statement

The animal study was reviewed and approved by the Institutional Animal Care and Use Committee (IACUC) of South China Agricultural University.

## Author Contributions

QX and WL: conceptualization. GS, S-bW, WL, and JW: methodology, software, and validation. SW, TC, and BZ: resources. WL and JL: writing – original draft preparation. YZ and QX: writing – review and editing. YZ and QX: funding acquisition. All authors have read and agreed to the published version of the manuscript.

## Conflict of Interest

The authors declare that the research was conducted in the absence of any commercial or financial relationships that could be construed as a potential conflict of interest.

## Footnotes

1 http://www.omicshare.com/tools

## Supplementary Material

Supplementary Figure 1 Identification of exosome special marker. Cell lysate: Whole cell lysate by RIPA. A-EXO, exosomes from adipose; M-EXO, exosomes from muscle.

Supplementary Figure 2 Validation of DE miRNAs change after treatment with exosome. NC, Adipocytes treated with PBS; M-EXO, Adipocytes treated with M-EXO. ***P* < 0.01.

Supplementary Figure 3 Integrated analysis of miRNAs – mRNA network. Green triangle: DE miRNA of M-EXO; Red triangle: DE miRNA of A-EXO; Blue circle: target gene of miRNA.

Supplementary Table 1 Information of primers.
