# Supplementary figures and images for: Comparative Analysis of MicroRNA Expression Profiles Between Skeletal Muscle- and Adipose-Derived Exosomes in Pig
Source: Front Genet. 2021 May 31;12:631230. doi: 10.3389/fgene.2021.631230 (PMC8202525; doi:10.3389/fgene.2021.631230)

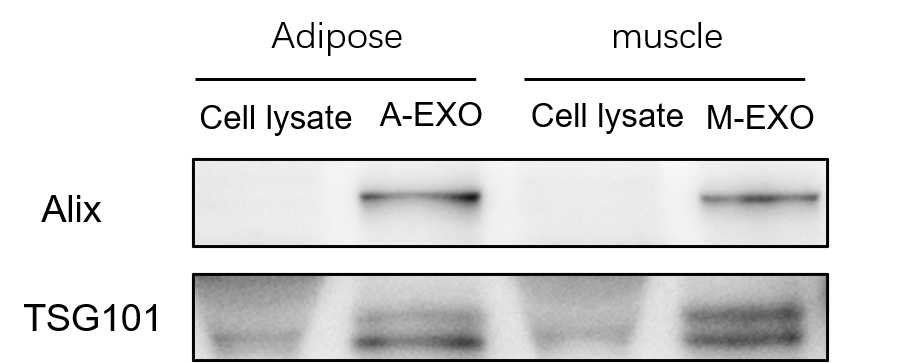

Supplement: Supplementary Figure 1 — Identification of exosome special marker. Cell lysate: Whole cell lysate by RIPA. A-EXO, exosomes from adipose; M-EXO, exosomes from muscle. [file Image_1.PNG]

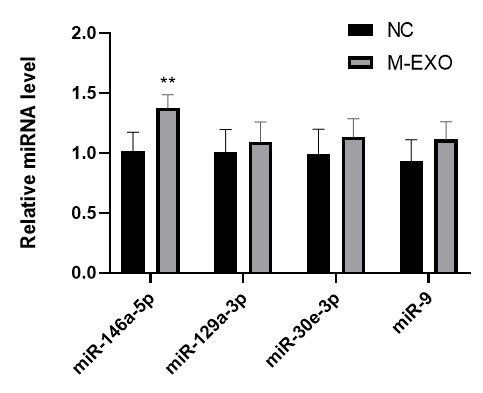

Supplement: Supplementary Figure 2 — Validation of DE miRNAs change after treatment with exosome. NC, Adipocytes treated with PBS; M-EXO, Adipocytes treated with M-EXO. **P < 0.01. [file Image_2.JPEG]

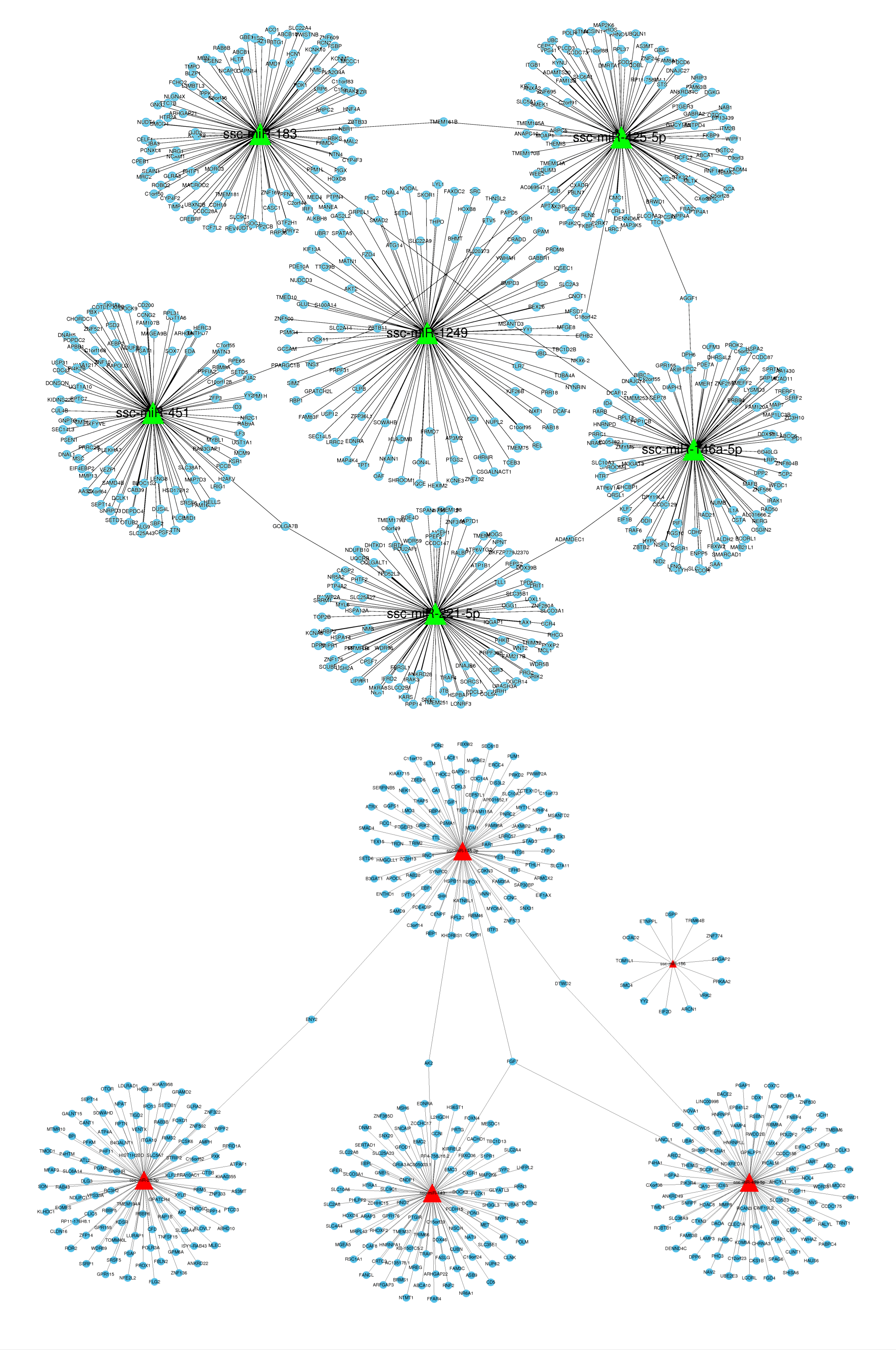

Supplement: Supplementary Figure 3 — Integrated analysis of miRNAs – mRNA network. Green triangle: DE miRNA of M-EXO; Red triangle: DE miRNA of A-EXO; Blue circle: target gene of miRNA. [file Image_3.TIF]
