# Supplementary material for: Comparative Analysis of MicroRNA Expression Profiles Between Skeletal Muscle- and Adipose-Derived Exosomes in Pig
Source: Front Genet. 2021 May 31;12:631230. doi: 10.3389/fgene.2021.631230 (PMC8202525; doi:10.3389/fgene.2021.631230)
Supplement: Supplementary Table 1 — Information of primers. [file Table_1.docx]

| **ID** | **RT primer** | **F/R primer** |
| --- | --- | --- |
| miR-146a-5p | CAGTGCAGGGTCCGAGGTCAGAGCCACCTGGGCAATTTTTTTTTTTVN | ATGCGCGCTGAGAACTGAATT  /CAGTGCAGGGTCCGAGGT |
| miR-129a-3p | GTCGTATCCAGTGCAGGGTCCGAGGTATTCGCACTGGATACGACATGCTTT | AACAGAAAGCCCTTACCCCAAAA  /CAGTGCAGGGTCCGAGGT |
| miR-125a | CAGTGCAGGGTCCGAGGTCAGAGCCACCTGGGCAATTTTTTTTTTTVN | AACCACTTCCCTGAGACCCTTTA  /CAGTGCAGGGTCCGAGGT |
| miR-24-3p | CAGTGCAGGGTCCGAGGTCAGAGCCACCTGGGCAATTTTTTTTTTTVN | AGTGGCATGGCTCAGTTCAG  /CAGTGCAGGGTCCGAGGT |
| miR-193a-3p | CAGTGCAGGGTCCGAGGTCAGAGCCACCTGGGCAATTTTTTTTTTTVN | AACAATTGGGTCTTTGCGGGC/CAGTGCAGGGTCCGAGGT |
| U6 | CGCTTCACGAATTTGTGTCAT | GCTTCGGCAGCACATATACTAAAAT  /CGCTTCACGAATTTGCGTGTCAT |
| CD36 | TTTTTTTTTTTTTTTTTT | GCAAAACGGTTGCAGGTCAA  /TGTACTGACAGTCCTGGGGT |
| PPARγ | TTTTTTTTTTTTTTTTTT | TGGTTGACACCGAGATGC /TGGAGTGGAAATGCTGGA |
| FABP4 | TTTTTTTTTTTTTTTTTT | AAGTCAAGAGCACCATAACC /TGATACATTCCACCACCAAC |
| C/EBPα | TTTTTTTTTTTTTTTTTT | TGAAAGAAGTGGGAGTGGGC /CTGGCCCAATTTGAAGGCAA |
| ACC | TTTTTTTTTTTTTTTTTT | CTGCACGGAAATGATCGCTG /GGTGAGGCGGTAACTGTTGA |

Table S1: Information of primers.
